# Quality evaluation of endometriosis guidelines using AGREE II

**DOI:** 10.1097/MD.0000000000031331

**Published:** 2022-10-28

**Authors:** Yan Lei, Xin Du, Dejun Chen, Yue Gao, Hongmei Lian

**Affiliations:** a Department of Gynecology, Maternal and Child Health Hospital of Hubei Province.

**Keywords:** AGREE II evaluation tool, endometriosis, expert consensus, guideline

## Abstract

To systematically evaluate the guidelines for endometriosis published in the past decade, and to provide reference for the selection of guidelines for endometriosis through quality evaluation and comparison.

**Methods:**

PubMed database, Embase database, evidence-based medicine clinical practice guidelines (CPG) database and the National Institute for Health and Clinical Excellence in the United Kingdom were searched by computer from December 2012 to December 2020 to retrieve published endometriosis CPG published by professional institutions or organizations. The search languages are English and Portuguese. Two researchers evaluated the quality of included CPG according to appraisal of guidelines for research and evaluation (AGREE II). The evaluation includes 6 areas: scope and purpose, participants, rigor of formulation, clarity of expression, applicability and independence. The recommendation level of CPG is determined by the distribution of standardized scores in the above 6 areas.

**Results:**

A total of 8 articles on endometriosis CPG were included, including 5 guidelines and 3 consensuses, covering 5 countries in 2 continents; the publication year was 2013 to 2020. The average standardized scores of the scope and purpose, participants, rigor, clarity, applicability and independence of CPG were 77.1%, 52.8%, 50.5%, 86.8%, 31.3%, and 36.5%, respectively. Among the 8 CPGs, 1 was grade A (recommended), 5 were grade B (recommended after improvement), and 2 were grade C (not recommended). Seven CPG recommendations were based on expert consensus, and one was developed through detailed literature retrieval, analysis and evidence rating evaluation. There was little difference between the guidelines in terms of treatment-related recommendations.

**Conclusions:**

The quality of endometriosis CPG released in 2013 to 2020 is quite different, and some CPGs are not ideal in terms of rigor, applicability and independence. The guidelines issued by NICE in 2017 are A-grade recommendations. The standardized scores in various fields are high, and the formation process of CPG is the most standardized, which is worth learning and reference.

## 1. Introduction

Endometriosis (endometriosis) refers to the occurrence, growth, infiltration, and repeated bleeding of endometrial tissue outside the uterus, which in turn leads to pain, infertility, nodules or masses. It is a common and frequently-occurring disease in women of childbearing age.^[1]^ Women with endometriosis usually have a series of pelvic abdominal pain symptoms, including dysmenorrhea, difficulty in intercourse, large amount of bleeding during menstruation, non-menstrual pelvic pain, ovulation pain, difficulty in urination and chronic fatigue.^[2]^ Endometriosis is also associated with infertility. There is a strong correlation between the severity of the disease and the impact on women’s fertility, due to impaired ovarian function, ovarian endometriosis cysts and subclinical pelvic inflammation, which may reduce the quality of oocytes and reduce the endometrial receptivity to implantation.^[3]^ As a common chronic gynecological disease requiring long-term management, the current guidelines and consensus related to endometriosis have been up to a hundred departments, which brings great difficulties to clinicians “decision-making and patients” informed choice. Therefore, it is very important for both doctors and patients to select high-quality guidelines or consensus as clinical diagnosis and treatment guidance.

Clinical practice guidelines (CPG) are designated and issued by professional academic organizations or departments based on the best evidence and combined with clinical practice to help clinicians and patients make clinical decision-making guidance, including guidelines, consensus, recommendation, etc, which can not only improve the diagnosis and treatment effect, but also make full use of medical resources.^[4]^ In this study, the internationally recognized guideline evaluation tool-appraisal of guidelines for research and evaluation (AGREE II) was used to evaluate the quality of the included CPGs through a comprehensive retrieval of foreign published CPGs specifically for endometriosis, so as to provide further reference for clinicians.^[5]^

## 2. Materials and Methods

### 2.1. Search strategy

PubMed database, Embase database, evidence-based medicine CPG database, National Institutes of Health and Clinical Medical Optimization were searched online from December 2012 to December 2020 with search terms of “endometriosis,” “guidelines,” “guidance,” “consensus.” We also searched the websites of guideline development organizations: Guidelines International Network Web site (http://www.g-i-n.net/), National Institute for Health for Health and Care Excellence website (https://www.nice.org.uk/guidance), National Guideline Clearinghouse (https://guidelines.gov/), Scottish Intercollegiate Guidelines Network (http://www.sign.ac.uk/), Clinical Practice Guidelines Portal website (https://www.clinicalguidelines.gov.au/), New Zealand Guidelines Group website (https://www.health.govt.nz/), BCGuidelines website (http://www.bcguidelines.ca/alphabetical), AQuMed Database website (http://www.aezq.de/aezq/publications). In addition, we searched Google Search Engine and checked the references of all the related guidelines to include more potential guidelines. All guidelines were based on previous published studies, thus no ethical approval and patient consent are required.

### 2.2. Inclusion and exclusion criteria

Inclusion criteria include: CPG for endometriosis published at home and abroad, developed or published by professional academic organizations or departments. Exclusion criteria include: old CPG issued by the same academic organization or department, lecture or expert review, review or research literature, guide interpretation, conference abstracts.

### 2.3. Literature screening and data extraction

Two researchers independently screened literature and extracted data according to inclusion and exclusion criteria and checked them. If disagreement arises, it shall be decided by the third researcher. Extraction of different CPG names (including guidelines, consensus and recommendations), topics, publication years, publication organization, guidelines development methods, evidence level evaluation methods and other basic information.

### 2.4. Quality evaluation included in CPG

Each included CPG was evaluated by 2 trained personnel for AGREE II. According to the “Instructions for Clinical Guidelines Research and Evaluation System II (Chinese version),” 23 entries in the following 6 fields were evaluated, including scope and purpose (3 entries), stakeholder involvement (3 entries), rigor of development (8 entries), clarity of presentation (3 entries), applicability (4 entries) and editorial independence (2 entries). Finally, a comprehensive evaluation was carried out according to the scores. Two researchers rated each item from 1 (very disagree) to 7 (very agree). Standardized scores in various areas = (actual rating-lowest possible rating)/(highest possible rating - lowest possible rating) × 100%. The higher the standardized score of a field, the more complete the reporting, reflecting the better the method of developing guidelines in the field. Combined with the scores of each field of the guide, the recommended level of the guide is divided into 3 grades, and grade A is recommended, that is, the standardized scores of the 6 fields are ≥60.00%; grade B is recommended after modification and improvement, that is, the standardized score of the field is <60.0%, and the standardized score of ≥3 fields is ≥30.0%; grade C is not recommended, that is, the number of fields with a standardized score of <30.0% in 6 fields was ≥3.

### 2.5. Evaluation quality control and statistical analysis

Before the formal evaluation, the 2 researchers used AGREE II to perform pre-experimental scoring on the randomly selected 2 guidelines to test the reliability of the results. The scoring results were statistically analyzed by SPSS 20.0 software, and the consistency of the evaluation results was investigated by intra-group correlation coefficient (ICC). When the ICC value is 0.81 to 1.00,^[6]^ it indicates that the consistency is high and formal experiments can be carried out.

## 3. Results

### 3.1. Literature retrieval process and included CPG

**Figure 1.**
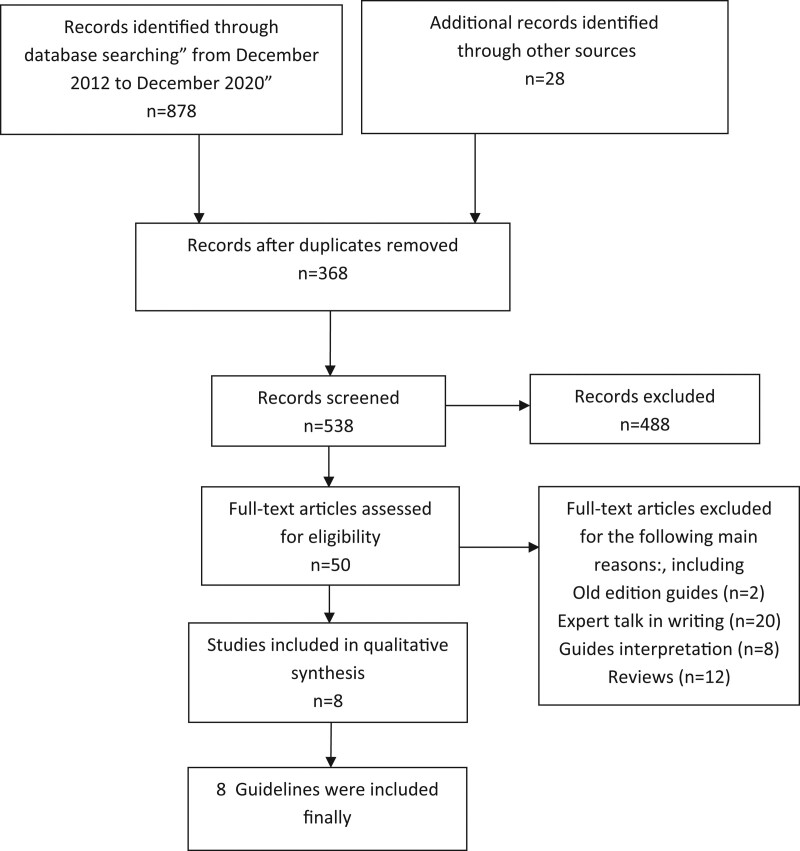
Literature search process and results.

A total of 906 relevant literature were obtained through database retrieval. According to the inclusion and exclusion criteria, 8 CPGs were finally included after excluding duplicate articles (Fig. 1).

### 3.2. Basic characteristics of incorporating CPG

Among the 8 CPGs included in this study, 5 guidelines and 3 consensus. Covering 5 countries of 2 continents; published in 2013 to 2018, with 6 first editions and 2 updated editions; 7 of them were conducted evidence quality rating and recommendation intensity evaluation based on the Grading of Recommendations (Assessment, Development and Evaluation, GRADE) method. In addition to the 2016 Portuguese guidelines, the 2017 WES endometriosis classification guidelines, and the 2018 Japanese guidelines, the remaining 5 CPGs are all about the diagnosis, treatment (drugs and surgery), and pain management of endometriosis in the reproductive system. Four articles also cover the treatment of patients with endometriosis and infertility. The 2014ESHRE guideline referred to the primary prevention of endometriosis, while the 2014 German guideline defined the secondary prevention of endometriosis and made recommendations (Table 1).

**Table 1:** General information of CPG.

Guideline	Published yr	Release organization	Country	Main theme	Version	Category
Neil et al, 2013^[7]^	2013	WES	—	Diagnosis, treatment, pain management, infertility treatment	First version	Consensus
G.A.J et al, 2014^[8]^	2014	ESHRE	—	Diagnosis, treatment, pain management, infertility treatment	Update	Guide
Ulrich et al, 2014^[9]^	2014	DGGG	Germany	Diagnosis, treatment, health care management, infertility treatment	First version	Guide
Carvalho et al, 2016^[10]^	2016	—	Portugal	medication	First version	Consensus
Neil et al, 2017^[11]^	2017	WES	—	Classification of endometriosis	First version	Consensus
NICE, 2017^[12]^	2017	NICE	Britain	Diagnosis, treatment, health care management, pain management	Update	Guide
Tetsuya et al, 2020^[13]^	2018	JSOG&JSE	Japan	Treatment of reproductive tract endometriosis	First version	Guide
Hyejin et al, 2018^[14]^	2018	KSE	Korea	Diagnosis, treatment, pain management, infertility treatment	First version	Guide

− = no such item, ESHRE = European association for human reproduction and embryology, DGGG = German society for gynecological sciences, GRADE = grading of recommendations, assessment, development and evaluation, JSOG & JSE = Japanese society of obstetrics and gynecology and Japanese society of endometriosis, KSE = Korean society of endometriosis, NICE = National institutes of health and clinical optimization, WES = world endometriosis society.

### 3.3. Quality evaluation results included in CGP

In this study, 2 evaluators used AGREE II to score the pre-experiment of the 2 randomly selected guidelines. The ICC value and 95% confidence interval of the pre-experiment results were 0.92 (0.82–0.97) and 0.91 (0.79–0.96), indicating that the consistency of the evaluation of the 2 researchers was good.

The average standardized scores for the scope and purpose, stakeholder involvement, rigor of development, clarity of presentation, applicability and editorial independence of the 8 CPGs included were 1 in grade A (recommendation), 5 in grade B (recommendation after improvement) and 2 in grade C (no recommendation), respectively (Table 2).

**Table 2:** Quality evaluation results of AGREE II domains incorporating CPG.

Guideline	Standardized scores in various areas(%)						Number of fields reaching different standardized scoring standards (number)			Recommendation grade
	Scope and purpose	Stakeholder involvement	Rigor of development	Clarity of presentation	Applicability	Editorial independence	Score > 60.0%	Score 30.0%~60.0%	Score < 30.0%	
Neil et al, 2013^[7]^	77.8	33.3	60.4	100	25.0	8.3	3	1	2	B
G.A.J et al, 2014^[8]^	94.4	83.3	62.5	100	20.8	100	5	0	1	B
Ulrich et al, 2014^[9]^	50.0	44.4	27.1	100	33.3	0	1	3	2	B
Carvalho et al, 2016^[10]^	55.6	22.2	43.8	88.9	16.7	0	1	2	3	C
Neil et al, 2017^[11]^	88.9	88.9	58.3	100	41.7	66.7	4	2	0	B
NICE, 2017^[12]^	100	100	87.5	100	100	66.7	6	0	0	A
Tetsuya et al, 2020^[13]^	66.7	50	50	61.1	0	0	2	2	2	B
Hyejin et al, 2018^[14]^	83.3	0	14.6	44.4	12.5	50	1	2	3	C
Average	77.1	52.8	50.5	86.8	31.3	36.5	—	—	—	—
Range	50.0	100	72.9	55.6	100	100	—	—	—	—

− = no such item, AGREE II = appraisal of guidelines for research and evaluation, CPG = clinical practice guidelines.

### 3.4. Composition analysis of endometriosis CPG

The formation and formulation of 7 endometriosis CPGs were based on the expert consensus, and their recommendations were finally decided by the expert vote. Although a large number of relevant literature retrieval and analysis were also carried out in the process of guideline formulation, many key issues were not answered due to the lack of high-quality and new research literature. CPG, which relies on detailed literature retrieval, analysis and evidence level assessment, is a guide issued by NICE in 2017.

Seven CPG statements used GRADE system as evidence level evaluation method. Six guidelines specify the number of recommendations or declarations recommended. All CPGs issued conflict of interest statements and provided references (Table 3).

**Table 3:** Composition of endometriosis CPG.

Guideline	Publishing organization	Evidence quality evaluation method	Main formulation methods	Number of declarations/ resolutions(number)	CPG pages	Number of references
Neil et al, 2013^[7]^	WES	GRADE	Experts consensus	69	17	128
G.A.J et al, 2014^[8]^	ESHRE	GRADE	Experts consensus	83	13	121
Ulrich et al,2 014^[9]^	DGGG	—	Experts consensus	—	15	211
Carvalho et al, 2016^[10]^	—	GRADE	Experts consensus	—	11	83
Neil et al, 2017^[11]^	WES	GRADE	Experts consensus	28	10	15
NICE, 2017^[12]^	NICE	GRADE	Literature search	21	363	256
Tetsuya et al, 2020^[13]^	—	GRADE	Experts consensus	8	26	164
Hyejin et al, 2018^[14]^	KSE	GRADE	Experts consensus	71		

− = no such item, CPG = clinical practice guidelines.

## 4. Discussion

Endometriosis is one of the common chronic diseases in gynecology. It is currently considered as a hormone-dependent disease, and its incidence accounts for 10% of the chronic diseases in women of childbearing age.^[15]^ The disease often leads to chronic pelvic pain, infertility and other diseases, which seriously affects the quality of life of patients.^[16]^ Its early diagnosis is difficult, and the treatment effect is poor.^[17,18]^ Therefore, high quality CPG should be followed in clinical decision making. The AGREE II system is universal and suitable for any disease area in all health care links. It can help health providers to evaluate themselves before they adopt the recommendations recommended by the guidelines. It can make the guideline developers follow a structured strict development method, help policy makers to determine which CPGs are suitable for practical applications, and also help health professionals to improve the ability to strictly evaluate the guidelines.^[19]^

From the 8 CPGs included in this study, AGREE II evaluation results showed that 1 was A grade recommendation, 5 were B grade recommendation and 2 were C grade recommendation. The average scores in fields 1 (scope and purpose) and 4 (clarity of presentation) were ≥60%, and the average scores in fields 5 (applicability) and 6 (editorial independence) were <30%, indicating that most of the CPGs for endometriosis did not pay enough attention to these 2 fields.

Main shortcomings: The average score of field 3 (rigor of development) was 50.5%, because the lack of high-quality and multi-center clinical research data led to the formulation of most CPGs based on clinical experience and consensus of experts; no detailed inclusion and exclusion criteria; there is no clear description of the method for forming recommendations; some CPGs do not even specify the strength and limitations of the evidence. The average score of filed 5 (applicability) was 31.3% because most CPGs did not mention the promotion and impediments to application; economic cost and health budget were not mentioned. The general score of field 6 (editorial independence) is not ideal. Although each CPG has a statement of interest conflict, the wording of most guidelines is not clear about the point that the views of sponsors do not affect the content of the guidelines. In particular, Japan’s CPG has barely elaborated on this field. Overall, the progress of endometriosis CPG in the past decade was limited, especially in the recommendation of diagnosis and treatment strategies.

The limitations of this study are as follows. The limitations of research tools: there are no primary and secondary divisions in the 6 fields of AGREE II, which leads to the fact that some CPG levels may not be consistent with the actual. In fact, the quality of GDP cannot be evaluated only by the level, which is also clearly pointed out in the use instructions of AGREE II. Language limitation: This study was limited by language, only English and Portuguese CPG were included, and no further study was conducted on CPG in other languages. AGREE II is only an evaluation system, which is subject to the subjective influence of design developers. It is not necessarily suitable for all regions and countries, and it needs to be refined and updated.

In this study, the diagnosis and treatment strategies and methods recommended by the 8 endometriosis CPGs were not significantly different, but in fact, many problems were controversial in the process of CPG production or in the diagnosis and treatment of patients with endometriosis.^[20]^ The 2017 NICE guide has high scores in 6 fields, and is the only CPG published on the basis of literature retrieval and evidence grading assessment. It provides a detailed description of economic and social costs, promotion and impediments, and provides a detailed comparative analysis of drug treatment costs, which is worthy of promotion and reference.

## Author contributions

**Conceptualization:** Yan Lei.

**Data curation:** Yan Lei, Xin Du.

**Formal analysis:** Yan Lei, Dejun Chen.

**Funding acquisition:** Hongmei Lian.

**Investigation:** Xin Du, Dejun Chen, Yue Gao.

**Methodology:** Yan Lei.

**Project administration:** Yue Gao, Hongmei Lian.

**Software:** Dejun Chen, Hongmei Lian.

**Supervision:** Yue Gao.

**Validation:** Yue Gao.

**Visualization:** Yue Gao.

**Writing – original draft:** Yan Lei.

**Writing – review & editing:** Xin Du, Hongmei Lian.
